# Assessing the Neurodevelopmental Impact of Fluoxetine, Citalopram, and Paroxetine on Neural Stem Cell-Derived Neurons

**DOI:** 10.3390/ph17101392

**Published:** 2024-10-18

**Authors:** Kimia Hosseini, Andrea Cediel-Ulloa, Mohamed H. AL-Sabri, Anna Forsby, Robert Fredriksson

**Affiliations:** 1 Department of Pharmaceutical Bioscience, Uppsala University, 751 24 Uppsala, Swedenrobert.fredriksson@uu.se (R.F.); 2 Department of Organismal Biology, Uppsala University, 752 36 Uppsala, Sweden; 3 Department of Surgical Science, Functional Pharmacology and Neuroscience, Uppsala University, 751 24 Uppsala, Sweden; 4 Department of Biochemistry and Biophysics, Stockholm University, 106 91 Stockholm, Sweden

**Keywords:** hNSCs, DNT, SSRIs, off-target

## Abstract

Background/Objectives: Many pregnant women globally suffer from depression and are routinely prescribed selective serotonin reuptake inhibitors (SSRIs). These drugs function by blocking the re-uptake of serotonin by the serotonin transporter (SERT) into neurons, resulting in its accumulation in the presynaptic cleft. Despite a large amount of research suggesting a potential link to neurodevelopmental disorders in children whose mothers took these drugs during pregnancy, their possible adverse effects are still debated, and results are contradictory. On the other hand, there is an immediate need for improved cell-based models for developmental neurotoxicity studies (DNT) to minimize the use of animals in research. Methods: In this study, we aimed to assess the effects of clinically relevant concentrations of paroxetine (PAR), fluoxetine (FLX), and citalopram (CIT)—on maturing neurons derived from human neural stem cells using multiple endpoints. Results: Although none of the tested concentrations of FLX, CIT, or PAR significantly affected cell viability, FLX (10 µM) exhibited the highest reduction in viability compared to the other drugs. Regarding neurite outgrowth, CIT did not have a significant effect. However, FLX (10 µM) significantly reduced both mean neurite outgrowth and mean processes, PAR significantly reduced mean processes, and showed a trend of dysregulation of multiple genes associated with neuronal development at therapeutic-relevant serum concentrations. Conclusions: Transcriptomic data and uptake experiments found no SERT activity in the system, suggesting that the adverse effects of FLX and PAR are independent of SERT.

## 1. Introduction

Depression stands as a widespread psychiatric disorder, impacting the lives of more than 300 million individuals globally. In addressing this condition, contemporary treatment guidelines advocate for the use of selective serotonin reuptake inhibitors (SSRIs) as the primary pharmacotherapeutic intervention and the first line of treatment for perinatal depression [1]. However, research on the safety and efficacy of employing this approach for managing depression during the periods of pregnancy and postpartum is scarce [2]. One of the main factors to take into consideration is that the effects of very early exposure to certain drugs, such as SSRIs, may not manifest immediately after birth; instead, they may become apparent at a later stage, posing a challenge for investigations [3]. There are several SSRIs available on the market, such as citalopram (CIT), fluoxetine (FLX), fluvoxamine, paroxetine (PAR), and sertraline [4]. Among them, citalopram stands out as the most selective 5-hydroxytryptamine (5-HT) uptake inhibitor, while paroxetine exhibits the highest clinical potency, according to existing research. In general, the rank order of selectivity remains consistent across in vitro, in vivo, and behavioral studies [5,6].

Several studies have discussed and/or linked in utero exposure to SSRIs and adverse infant outcomes. These outcomes include conditions such as adaptation syndrome [7], low birth weight [8], congenital anomalies [9], and implications for both motor and cognitive disorders [10]. The potential associations between SSRI intake by pregnant mothers and neurodevelopmental disorders such as autism spectrum disorder (ASD) have been the focus of several studies, including clinical, in vivo, and in vitro studies [11,12,13,14,15,16,17,18]. However, the majority of the data available are inconclusive and have led to contradictory opinions regarding SSRI use during pregnancy.

Nevertheless, amid this uncertainty, these findings suggest that neurodevelopmental outcomes may represent a sensitive endpoint in humans after in utero exposure [19].

SSRIs constitute a class of antidepressants believed to exert their primary effects through the inhibition of the 5-HT transporter in pre-synaptic serotonergic neurons, leading to an increased extracellular availability of serotonin. It is noteworthy that various SSRIs have been claimed to interact with alternative neurotransmitters, extending beyond the serotonergic system [20,21,22,23]. Furthermore, serotonin regulates a broad spectrum of physiological functions through its interaction with various receptors named 5-HT1–5-HT7 [24].

FLX, in particular, is one of the most common antidepressants that is prescribed to the general population as well as pregnant women [25]. Research findings indicate an accumulation of FLX in the human brain compared to plasma. This results in a brain-to-plasma ratio of approximately 20:1 [26,27]. There are also several studies showing that FLX at different concentrations induced and prevented cell death [28,29,30].

The high maintenance, relevance, and time-consuming nature of animal tests for neurodevelopmental studies have raised concerns among experts. Therefore, there is a recommendation to use in vitro alternatives of human origin to minimize and eventually eliminate the use of animals in DNT studies [31,32,33]. Human neural cultures provide a relevant model for studies on neuronal development, owing to their alignment with human biology and their capacity for differentiation, migration, and maturation. Additionally, they address the ethical concerns associated with animal research, following the principles of the 3Rs (Replacement, Reduction, and Refinement).

This study builds upon prior research, where we demonstrated the successful differentiation of human embryonic stem cells (hESCs) into human neural stem cells (hNSCs) [34]. Additionally, we characterized maturing neurons at various time points using Ion AmpliSeq technology; the culture was identified as a co-culture of astrocytes and neurons, with GABAergic neurons being the predominant neuronal phenotype (unpublished data [35]) and not expressing SERT. However, given that some studies suggest SSRIs can also have off-target effects independent of SERT [36], we aimed to investigate the impact of FLX, CIT, and PAR on viability, differentiation, and maturation of hNSCs to these neurons. For this, we exposed our cultures to different concentrations of these SSRIs for 10 days to stimulate exposure during early brain development. Effects were assessed with a multidimensional approach, including viability, RT-qPCR, neurite outgrowth, and apoptotic assays.

## 2. Results

### 2.1. Effect of SSRIs on Cell Viability

**Figure 1:**
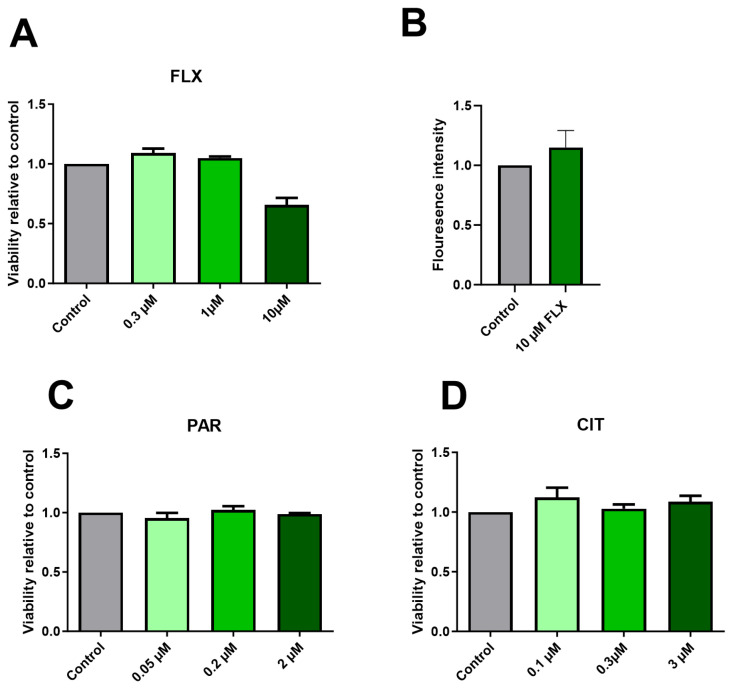
Effects of SSRIs on cell viability in maturing neurons after 10 days of exposure. Graph (**A**) illustrates a tendency toward reduction in neuronal viability following 10-day exposure to FLX 10 µM, and (**B**) shows increased caspase activity at 96 h post-exposure to FLX 10 µM compared to the control. Graphs (**C**,**D**) show no obvious changes in the viability of the neuronal culture compared to the control when exposed to PAR and CIT, respectively. Viability assays were performed in 3–4 independent experiments, and apoptosis assays were performed in 2 independent experiments. There was no statistically significant change compared to the control.

In this study, we investigated the impact of exposure to CIT, FLX, and PAR at varying concentrations over 10-day periods on neuronal differentiation. Cell viability was assessed using the Alamar Blue reagent. Figure 1C,D show no notable reduction in viability following exposure to PAR and CIT across all concentrations tested. However, exposure to FLX 10 µM shows a reduction in the viability of the culture even though it was not found to be statistically significant (Figure 1A).

Caspase activity was measured to determine if the tendency towards a reduction in viability caused by FLX 10 µM was associated with apoptosis. The caspase assay was performed after 96 h post-seeding and culture exposure to FLX 10 µM with 2–3 technical replicates (2 independent experiments). The result (Figure 1B) suggests an increased caspase activity when the exposed cells are compared to the control, which may point to an underlying mechanism. It is worth noting that increasing the number of independent replicates could potentially influence the outcome and enhance the likelihood of observing a significant reduction in viability and a significant increase in caspase activity. However, one single result from an apoptosis experiment including FLX (20 µM) shows increasing caspase activity with increasing concentration (Supplementary Figure S1).

### 2.2. Paroxetine and Fluoxetine Demonstrate a Reduction in Neurite Outgrowth

**Figure 2:**
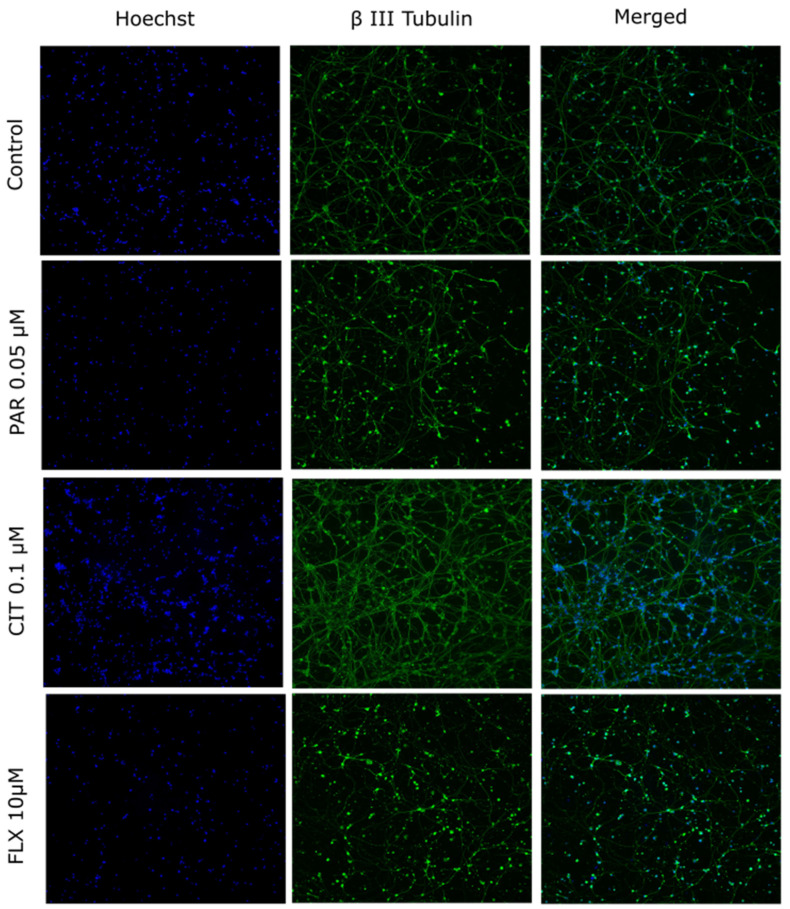
Representative images of neurons. Cells were exposed to DMSO 0.1% (control), PAR 0.05 µM, CIT 0.1 µM, or FLX 10 µM. Nuclei are stained with Hoechst 33342 and visualized in blue. Neurons were stained against the BIII tub and are visualized in green.

**Figure 3:**
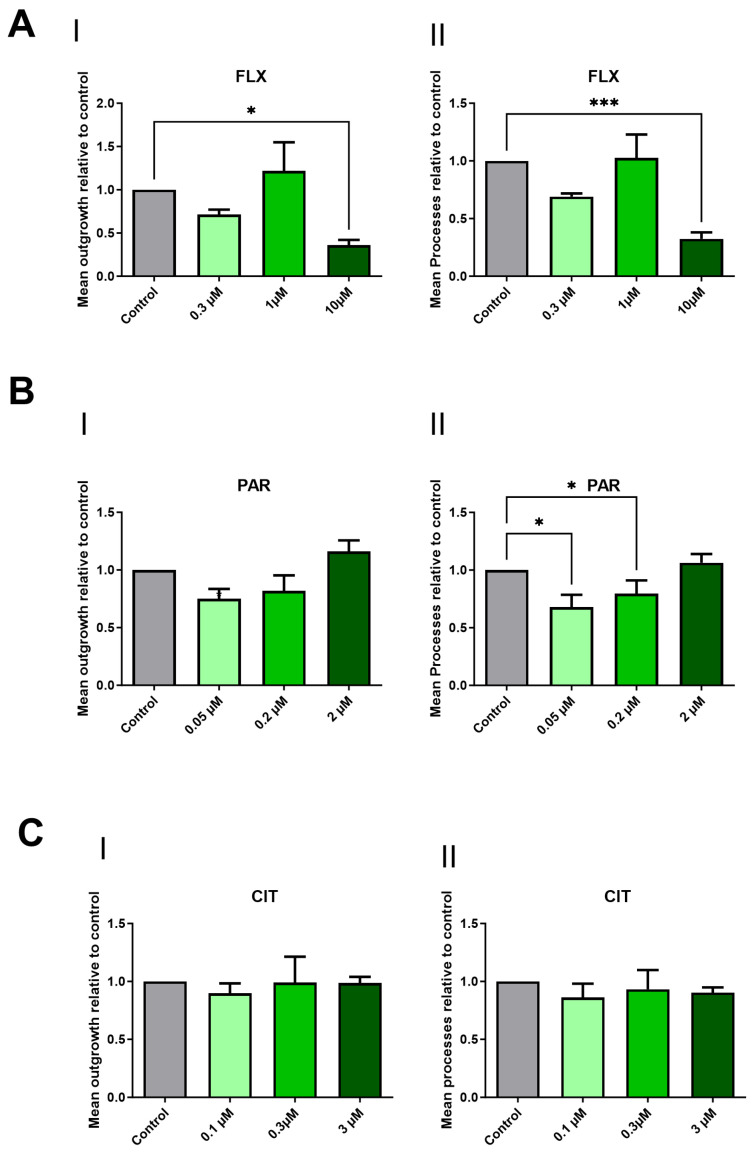
Effects of the SSRIs on neuronal morphology. Mean outgrowth and mean processes were determined following 10 days of exposure to FLX (**A**), PAR (**B**), and CIT (**C**). Graphs are presented as a fraction of the control of outgrowth parameters relative to the control. Neurite outgrowth assays were performed in 3–4 independent experiments. Statistically significant differences compared to the control group are shown by *p* < 0.05 (*) and *p* < 0.001 (***).

The impact of FLX, CIT, and PAR on neurite outgrowth in maturing neurons was assessed. The assessment of DNT endpoints is typically conducted at non- or minimally cytotoxic concentrations [37]. Despite its impact on viability, FLX was included at a concentration of 10 µM for neurite outgrowth analysis. This decision was based on the fact that FLX accumulates in the brain, reaching therapeutic concentrations of up to 20–30 µM. Thus, selecting a concentration of 10 µM, which exceeds plasma concentration, maintains clinical relevance. Figure 2 shows representative microscopic images of cells exposed to DMSO 0.1% (control), PAR 0.05 µM, CIT 0.1 µM, or FLX 10 µM. The mean neurite outgrowth and mean processes exhibited a statistically significant reduction when cells were exposed to FLX 10 µM (Figure 3A) and mean processes in the case of PAR 0.05 µM and 0.2 µM (Figure 3B(II)). Citalopram, on the other hand, did not show any notable change in the parameters at any concentrations (Figure 3C).

Following the selection of varying concentrations of CIT, FLX, and PAR, coupled with viability assays and subsequent neurite outgrowth analysis, PAR and FLX were chosen for RT-qPCR analysis at their therapeutic relevant concentrations to explore potential molecular effects.

Moreover, FLX at 10 µM, a concentration higher than therapeutic plasma but still lower than the estimated brain concentration according to existing literature [26], was specifically selected for apoptotic measurement assays. This decision was motivated by the interest in comprehensively understanding the apoptotic response at this clinically relevant concentration in this culture.

### 2.3. Effect of FLX and PAR on 21 Neurodevelopmental-Associated Transcriptional Markers

To assess the potential neurotoxicity of FLX and PAR at the mRNA level, a comprehensive analysis of 21 genes was conducted. The genes selected for this study were chosen based on their critical roles in key neuronal processes, including differentiation, maturation, and signaling. These processes are fundamental to proper neural development, and any disruption to them may result in adverse neurodevelopmental outcomes. A summary of the functions of each gene is provided in Table S2 Supplementary Data. Additionally, where available, their link to neurodevelopmental disorders is mentioned. The cells were exposed during differentiation and maturation to therapeutic concentrations of the drugs for 10 days, and subsequent gene expression levels were evaluated using RT-qPCR after harvesting the cells.

**Figure 4:**
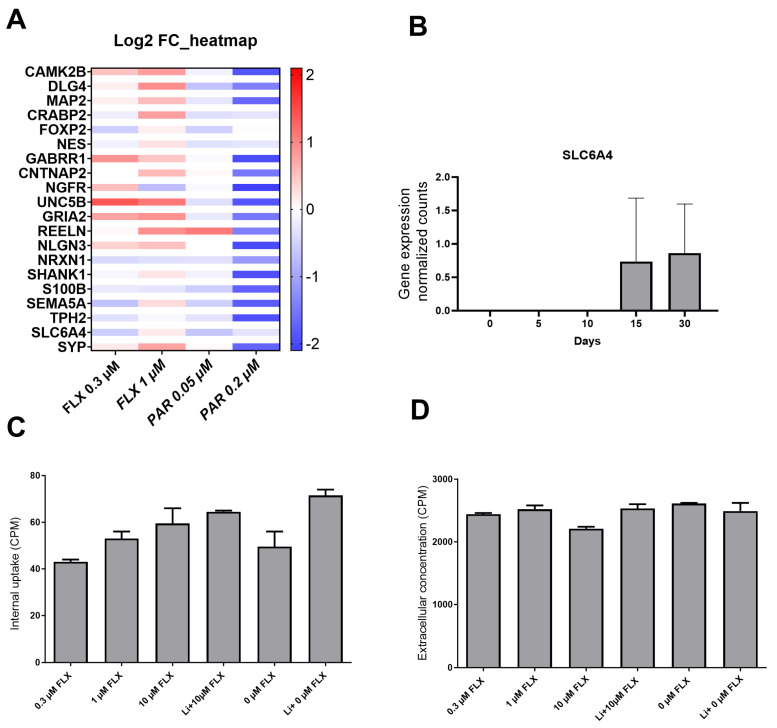
Absence of SERT activity and transcript. (**A**) Relative mRNA levels (Log2 fold change) of 21 genes measured by RT-qPCR after 10 days of exposure to FLX or PAR compared to unexposed cells. (**B**) mRNA levels of SERT (SLC6A4, normalized counts) during differentiation, analyzed by AmpliSeq. (**C**) Cellular uptake of ^3^H-5HT during 90 min background signal was removed during scintillation counting, and (**D**) extracellular ^3^H-5HT in CPM. RT-qPCR and AmpliSeq experiments were performed in 3–5 independent experiments, and uptake experiments were performed in 2 independent experiments. There was no statistically significant change compared to the control.

A trend of reduction in the expression level of multiple genes was observed when the cells were treated with PAR 0.2 µM, although it was not statistically significant. No notable changes were observed in the other tested conditions. Importantly, the marker SLC6A4 (SERT) consistently exhibited Threshold Cycle (Ct) values in the range of 32–34, which is the background level in the assay, remaining unaffected across all exposure conditions, indicating no or negligible expression of this transporter (Figure 4A). The lack of expression of SERT (gene *SLC6A4*) was confirmed by AmpliSeq analysis performed at various time points during maturation in our cell model. However, a slight increase in *SLC6A4* expression was observed on days 15 and 30 during maturation. (Figure 4B). These results confirm negligible to zero expression of SERT during the exposure and culture period up to differentiation day 10.

### 2.4. 3H-5HT Uptake Assay Indicated the Absence of SERT

Cells, after 10 days of differentiation and maturation, were exposed to increasing concentrations of FLX (0.3 µM, 1 µM, and 10 µM) and 3H-5HT for 1 h. Extracellular concentration and intracellular uptake of 3H-5HT were measured and compared to the control cells without FLX. According to graphs 4C and 4D, FLX did neither inhibit the cellular uptake in sodium-containing nor sodium-excluding buffers at any concentration. The reason to exclude sodium was to see if the uptake would be different, as transport via SERT is sodium-dependent [38]. Since there was no observed change or decrease in the uptake, it is assumed that there is no SERT activity in this cell model.

## 3. Discussion

One of the key processes involved in neurodevelopment is neurite outgrowth. If disrupted, the irreversible effects on neuronal connectivity can manifest later in life. Neurite outgrowth serves as a crucial endpoint in analyzing substances for DNT, and multiple protocols have been developed to enhance its readout [37,39,40,41,42].

In this study, we used our maturing neurons derived from human NSCs as a 2D in vitro tool to explore the neurodevelopmental impact of CIT, FLX, and PAR. Based on previous studies, SSRIs such as FLX have been found to affect the development of neurons, the associated processes, cell viability, and embryonic body morphogenesis regardless of SERT expression [36,43,44,45]. In our analysis, which employed various approaches, we confirmed SERT is not expressed in this cell line. Therefore, we sought to determine whether different SSRIs could induce any effects in our culture.

The culture was exposed to different concentrations of the drugs for a total duration of 10 days. Our goal was to maintain the culture for as long as possible, but we found out that with more than 10 days, the extensive neurite network formation makes it difficult to accurately measure outgrowth using our tool of analysis, potentially compromising the reliability of the results. Therefore, we opted for day 10, when the neurite networks had formed but not to the extent that they interfered with the analysis.

According to our results, there was no statistically significant viability change from any of them, but FLX, at 10 µM, affected the cell viability. Reports are showing different effects on cell viability after exposure to FLX. For instance, in a study involving mesencephalic and striatal rat cells, exposure to FLX at concentrations up to 15 μM resulted in a slight increase or no effect in cell viability. However, in the same study, the viability of hippocampal and cortical cells decreased when exposed to FLX at concentrations of 2.5 μM and higher [45]. FLX exposure to neural progenitor cell cultures of rat origin showed increased cell proliferation at lower concentrations and decreased proliferation of neutrospheres at the highest concentration of 20 µM [46]. Due to the viability reduction by FLX (10 µM) in our model, we performed a caspase 3/7 assay on cells that were exposed to it, which showed a tendency to increase in caspase activity after 96 h exposure based on 2 independent experiments having consistent results. Apoptosis signaling, induced by FLX, was found to occur through extrinsic/intrinsic pathways in vitro [47].

Following the neurite outgrowth assay, it was discovered that FLX, at 10 µM, caused a statistically significant reduction in the mean neurite outgrowth and mean process parameters. In a study by [48], they showed that FLX at 1 µm significantly potentiated neurite outgrowth in PC12 cells, but in another study, FLX at 5 µM was found to suppress the neurite outgrowth in neuro2a neuroblastoma cells [49]. These contradictory results from both viability and outgrowth studies indicate that factors such as cell type, exposure time, and concentration can indeed lead to varying outcomes, highlighting the importance of further studies involving a range of relevant cell cultures that can represent human development, extended exposure times, and also clinically relevant concentrations. Employing multiple approaches will also help to clarify developmental effects more precisely.

Analysis of the outgrowth assay also revealed that the culture exposed to PAR at 0.05 and 0.2 µM induces a statistically significant reduction in mean processes for both concentrations. Furthermore, the q-PCR experiment showed that while exposure to PAR at 0.2 µM caused a lower expression of several neurodevelopmental-associated genes, this effect was not statistically significant. Nonetheless, several of these genes, such as microtubule-associated protein 2 (*MAP2*) [50], neuroligin 3 (*NLGN3*) [51,52], and synaptophysin (*SYP*) [53], are involved in the development of neurites such as axons or synaptogenesis. The results obtained from the outgrowth assay after PAR exposure are consistent with a study utilizing brain spheres derived from human iPSC as their in vitro model, in which outgrowth inhibition was also observed at relevant therapeutic concentrations [54]. Although they used a cell line shown to express *SLC6A4* (quantified by q-PCR), they also observed adverse effects of PAR similar to our cell model, which lacks SERT. This also suggests that PAR may have neurodevelopmental toxicity regardless of SERT expression. Additionally, because it can cross the placenta [55], it is crucial to investigate its potential for developmental toxicity in multiple aspects.

Our results also show that 0.05 and 0.2 µM of PAR decreased mean processes, whereas 2 µM, which was the highest tested concentration, did not influence any measurement, suggesting a non-monotonic dose response. Atypical dose responses have previously been seen with antidepressants, including SSRs. Several studies have also shown that SSRIs can induce effects in a non-monotonic dose–response fashion [56,57,58]. However, in our case, this observation was based on only three concentrations, and to certainly establish a non-monotonic dose–response relationship, additional studies with a broader range of concentrations are needed.

Based on the RT-qPCR, AmpliSeq, and supportive results from the uptake assay, there is no SERT expression or activity in our cell culture model. Interestingly, a slight increase in 3H-5HT uptake with increasing concentrations of FLX was observed. This is not the expected effect of FLX on SERT transport. A decreased uptake of 3H-5HT would be expected in the presence of FLX, being an SSRI. This indicates that SERT is not functional in our neuronal cultures. Moreover, there are studies suggesting that SSRIs can have certain effects on the development of neurons irrespective of SERT expression [43]. For example, FLX has been found to have an inhibitory effect on voltage-gated potassium channels in human embryonic kidney (HEK) cells [44]. Therefore, the effects observed in our study must be mediated by the drugs acting on off-target molecules in the cells. This could also explain why there is such a difference in effect from the different SSRIs tested here because even if all have the 5-HT transporter as their primary target, the off-targets can be different for the different SSRIs. Adverse effects on synaptic transmission have been seen on both serotonergic and non-serotonergic neurons [59], but we cannot rule out that the lack of expression of SERT could have been a reason for the neurotoxic effects observed in our study. Given that SSRIs may have off-target effects, future developmental toxicity studies on SSRIs should expand their focus beyond neuronal cells. If SSRIs can influence cells in the absence of SERT [36], it raises the possibility that these drugs might similarly affect other cell types in the developing fetus, whereas SERT is not abundantly expressed.

## 4. Materials and Methods

Reagents

H9 cell line (WiCell, Madison, WI, USA), Poly-L-ornithine hydrobromide (PLO) (Sigma-Aldrich Cat. No. P4957, St. Louis, MO, USA), laminin (Sigma Aldrich Cat. No. L2020), PSC neural induction medium (Thermo Fisher Scientific Cat. No. A1647801 Waltham, MA, USA), advanced DMEM/F-12 (Thermofisher Scientific Cat. No. 12634010), STEMdiff™ Forebrain Neuron Differentiation Medium (Stem Cell Technology Cat. No. 08600, Cambridge Research Park, Waterbeach, UK), STEMdiff™ Forebrain Neuron Maturation medium (Stem Cell Technology Cat. No. 08605), CIT (Sigma Aldrich Cat. No. Y0001007), FLX (Sigma Aldrich Cat. No. F0253000), PAR (Sigma Aldrich Cat. No. PHR1804), Alamar Blue (Thermo Fisher Scientific Cat No. DAL 1025), βIII-tubulin (Abcam Cat. No ab 18207 Cambridge, UK), SYBR Green Supermix (Bio-Rad Cat. No. 1725270 Hercules, CA, USA), Caspase 3/7 working solution (Stem cell technology Cat. No. 100-0920), iScript reverse transcription super mix (Bio-Rad Cat. No. 1708840), Hoechst- (Thermofisher Scientific Cat. No. 33342), Aurum TM Total RNA Mini Kit (Bio-Rad Cat. No. 7326820).

### 4.1. Cell Culture

hNSCs differentiated from H9 cells based on our previous publication [34] were initially seeded onto double-coated plates with Poly-L-ornithine hydrobromide (PLO) and laminin at a density of 6 × 10 cells/cm^2^. Following overnight incubation in neural expansion medium (Neural induction medium+ advanced DMEM-F12 and gibco neural induction supplement) at 37 °C with 5% CO_2_, the culture medium was replaced the next day with STEMdiff™ Forebrain Neuron Differentiation Medium.

After two days, the hNSCs were sub-cultured at a ratio of 3 × 10^4^ cells/cm^2^ onto the previously coated PLO/Laminin culture plates in STEMdiff™ Forebrain Neuron Maturation medium. Notably, this maturation medium was supplemented with varying concentrations of PAR, CIT, and FLX. Throughout the 10-day culture period, the medium, including freshly diluted SSRIs, was replenished every 2–3 days to ensure optimal conditions for neural maturation.

### 4.2. Drug Concentrations and Exposure

During the exposure phase, cells were exposed to CIT at 0.1 µM, 0.3 µM, and 3 µM; FLX at 0.3 µM, 1 µM, and 10 µM; and PAR at 0.05 µM, 0.2 µM, and 2 µM. The two lowest concentrations are therapeutically relevant and fall around the range reported in existing literature for plasma levels [54,60,61,62,63,64]. Additionally, we included a concentration 10 times higher than the plasma levels.

All drugs were initially dissolved in dimethyl sulfoxide (DMSO) and stored as stock solutions at −20 °C at most for 1 month. To avoid freezing and thawing, several aliquots were prepared. At the time of exposure, new serial dilutions of each drug were prepared in the culture medium to achieve the desired concentrations. The final concentration of DMSO in the medium was consistently maintained at 0.1% to minimize any potential solvent-related effects. As a solvent control, cells were also exposed to 0.1% DMSO to account for any non-specific effects of the solvent.

### 4.3. Cell Viability Assay Using Resazurin Assay (Alamar Blue)

A cell viability assay using the resazurin method was performed. Cells were seeded onto 96-well plates at a density of 3 × 10^4^ cells/cm^2^ and exposed to various drug concentrations at the time of plating, or only 0.1% DMSO as a control. After a 10-day culture period, the spent medium was replaced with 10 µL of Alamar Blue diluted in 90 µL of fresh medium.

Subsequently, the plates underwent a 1 h incubation at 37 °C in a cell culture incubator, protected from direct light. The reduction in resazurin was quantified by measuring fluorescence (excitation 535 nm, emission 595 nm) using a plate reader (Tecan Infinite F200, Männedorf, Switzerland).

### 4.4. Apoptosis Detection Assay

Cells were seeded in 96-well plates, maintaining the same cell density as in the cell viability assay. They were exposed to FLX (10 µM) for the duration of 96 h. Following the exposure period, 100 µL/well of caspase 3/7 working solution was added to all wells. The plate was incubated for 1 h at 37 °C, and fluorescence at Ex/Em 490/525 nm was measured using a plate reader (Molecular Devices, San Jose, CA, USA) to assess caspase activation as a hallmark of apoptosis.

### 4.5. Analysis of Neurite Outgrowth

Neurite outgrowth measurement was conducted on clear-bottom, black 96-well plates (Corning Cat. No. 3340) coated with PLO/Laminin, employing the same density, culture duration, and exposure procedure. The chosen concentrations mirrored those in the cell viability assay. The cells were stained with 1 µM Hoechst-33342 in the exposure medium, followed by 30 min of incubation at 37 °C and 5% CO_2_. In the subsequent step, cells were fixed with 4% paraformaldehyde, washed twice with PBS, and stored at −4 °C until staining. βIII-tubulin (1 µg/mL) (Abcam Cat. No. ab6142) was used to stain the neurons as the primary antibody, followed by the corresponding secondary antibody. Images were captured utilizing a high-content ImageXpress fluorescence microscope (Molecular Devices, USA) equipped with a 10X objective lens. To comprehensively cover each well, the imaging process was systemically conducted at nine different sites, resulting in the acquisition of nine images per well and channel [65].

The total number of cells was quantified using Hoechst 33342 staining, with nuclei identified in the DAPI channel. Cell bodies were identified with a maximum width of 75 µm, while outgrowths had a maximum width of 2 µm. Mean neurite outgrowth and mean processes were calculated using the Neurite Outgrowth plug-in in the MetaXpress^®^ Software Mix 6.6.3 (Molecular Devices) [66], as the software can perform multi-parameter analysis.

### 4.6. RNA Extraction and cDNA Synthesis

Aurum TM Total RNA Mini Kit was used to extract DNA-free total RNA from lysed neurons, according to the manufacturer’s protocol. The extracted RNA was eluted in 40 µL of nuclease-free water and subsequently stored at −80 °C for later analysis. The quality and concentration of RNA were assessed using TapeStation (Agilent Technologies, Santa Clara, CA, USA), measuring the RNA Integrity Number (RIN), and cDNA synthesis was performed using the iScript reverse transcription super mix followed by diluting in RNAse-free water and stored at −80 °C until the experiment.

### 4.7. Reverse Transcription-Quantitative Polymerase Chain Reaction Analysis of 21 Genes Associated with Neurodevelopmental Toxicity or Neuronal Activity

Following the acquisition of results from the cell viability and neurite outgrowth assays, a decision was made to investigate the impact of PAR and FLX at two therapeutic-relevant concentrations. Their effect on 23 genes, including two housekeeping genes (Supplementary Table S1), was analyzed using RT-qPCR. The 21 selected genes are all involved in neural development and have been linked to developmental disorders based on published studies. Primer pre-casted 384-well plates were acquired from Bio-Rad. According to the Bio-Rad PrimePCR instruction manual, 10 ng of cDNA in 10 µL volume was added to each well. Each gene was subjected to 3–4 biological replicates, each performed in 2 technical replicates. The reactions were executed according to the manufacturer’s instructions using a CFX384 Real-Time PCR Detection System (Bio-Rad, Hercules, CA, USA) using SsoAdvanced Universal SYBR Green Supermix.

### 4.8. AmpliSeq Analysis of Maturing Neurons without Exposure

A comprehensive transcriptomic investigation of maturing neurons at different time points (days 0, 5, 10, 15, and 30) was previously conducted according to the instruction (Thermofisher Scientific, Waltham, MA, USA) and was normalized by DESeq2 [67] using R4.3.1 (R Core Team, 2023). For the current study, we focused on serotonin 5-HT transporter (*SLC6A4*) expression at five different time points using three-five independent experiments. Data analysis from AmpliSeq is available from the corresponding author upon reasonable request.

### 4.9. Uptake Experiments to Measure SERT Activity

The uptake assay was performed essentially as previously described [68]. Briefly, cells cultured on 24 well plates were treated with 0.3 µM, 1 µM and 10 µM 3H-5HT in KRH buffer (118 mM NaCl, 4.8 mM KCl, 1.2 mM MgSO_4_, 2.5 mM CaCl_2_, 10 mM HEPES, adjusted to pH 7.8) for 60 min. FLX 10 µM in Li buffer (118 mM LiCl, 4.8 mM KCl, 1.2 mM MgSO_4_, 2.5 mM CaCl2, 10 mM HEPES, adjusted to pH 7.8) were also added in the experiment to see if the uptake is affected independently of sodium. All concentrations were used in triplicates and the volume used was 200 µL. After incubation with 3H-5HT, the cells were washed three times with cold KRH buffer and Li buffer accordingly and lysed in 10% SDS in PBS. The lysates were then transferred to scintillation tubes containing scintillation solution (Zinsser Analytic, Eschborn, Germany) and counted using a Tri-Carb^®^ 4910 TR scintillation analyzer (Perkin Elmer, Waltham, MA USA). In addition, 10 µL of the KRH/Li buffer containing only 3H-5HT without FLX was counted as a control to measure concentration outside the cells. In addition, 10 µL of the extracellular solution was counted, and the numbers were adjusted for normalization to compensate for the smaller volume by multiplying with 20.

### 4.10. Statistical Analysis and Data Visualization

Statistical analysis was performed using GraphPad Prism software version 10.1.0 (San Diego, CA, USA) For viability and neurite outgrowth measurements, the results from the plate reader and ImageXpress were normalized against the control, and the statistical analysis was performed on the average normalized value of each experimental group and compared to the average of the controls (set = 1) employing non-parametric Kruskal–Wallis followed by an uncorrected Dunn’s multiple comparison test, and. The significance levels were set to *p* < 0.05 (*).

For RT-qPCR analysis, results were normalized against two housekeeping genes, and the statistic was performed on ∆Ct values using an unpaired parametric multiple comparison *t*-test. The results are presented in a heatmap as fold changes (2^−∆∆Ct^) as described by [69]. RT-qPCR, AmpliSeq, cell viability, and neurite outgrowth were performed in 3–5 independent experiments. Unless there was a technical error, all the measurements used in the analysis were utilized, which included 2–5 technical replicates. The results were presented in graphs and visualized by using the mean values of independent replicates with error bars representing the standard error of the mean (SEM).The uptake experiment and caspase assay were performed twice with 2–3 technical replicates with consistent results. For the caspase assay experiment, the results were normalized against the control (Set = 1). Graphs for uptake assays are plotted with representative data from one experiment.

## 5. Conclusions

In this study, the impact of clinically relevant concentrations of CIT, fluoxetine FLX, and PAR on non-serotonergic maturing neurons derived from human neural stem cells was assessed. The data revealed that FLX at 10 µM—still below the levels typically accumulated in the brain—and PAR at concentrations of 0.05 and 0.2 µM inhibited neuronal morphology parameters. Also, PAR exhibited a trend toward reduced expression of several neurodevelopmental-associated genes. These findings highlight the need for further research to evaluate the pharmacological safety of these antidepressants on fetal development when used during pregnancy.

## Data Availability

The original contributions presented in the study are included in the article/Supplementary Materials, further inquiries can be directed to the corresponding author.

## Supplementary Materials

The following supporting information can be downloaded at: https://www.mdpi.com/article/10.3390/ph17101392/s1, References [70,71,72,73,74,75,76,77,78,79,80,81,82,83,84,85,86,87,88,89,90,91,92,93,94,95,96,97,98,99,100,101,102,103,104] are cited in the Supplementary Materials. Table S1. Detailed information on the PrimePCR assay for the 21 selected genes associated with neural development, including 2 housekeeping genes, purchased from Bio-Rad. The PrimePCR design was performed using the Bio-Rad webpage (https://commerce.bio-rad.com/en-us/prime-pcr-assays/select-plate-template, accessed on 7 September 2023). Table S2. Summary of the function of the genes selected for RT-qPCR and their known association with developmental disorders. Figure S1. Caspase activity assay showed increased caspase activation in response to FLX treatment at 10 µM and 20 µM compared to the control demonstrating a concentration-dependent increase in caspase activity with FLX treatment after 96 h (Data represent the average of each group, derived from a single experiment with three technical replicates).

## Abbreviations

5-HT	5-hydroxytryptamine
ASD	Autism spectrum disorder
cDNA	Complementary DNA
CPM	Count per minute
CIT	Citalopram
DMSO	Dimethyl sulfoxide
DNT	Developmental neurotoxicity
FC	Fold changes
FLX	Fluoxetine
hESCs	Human embryonic stem cells
hNSCs	Human neural stem cells
PAR	Paroxetine
RT-qPCR	Reverse transcriptase quantitative polymerase chain reaction
SERT	Serotonin transporter
SSRI	Selective serotonin reuptake inhibitors
