# Supplementary material for: Assessing the Neurodevelopmental Impact of Fluoxetine, Citalopram, and Paroxetine on Neural Stem Cell-Derived Neurons
Source: Pharmaceuticals (Basel). 2024 Oct 18;17(10):1392. doi: 10.3390/ph17101392 (PMC11510426; doi:10.3390/ph17101392)

Supplementary Table S1. Detailed information on the PrimePCR assay for the 21 selected genes associated with neural development, including 2 housekeeping genes, purchased from Bio-Rad. The PrimePCR design was performed using the Bio-Rad webpage (www.commerce.bio-rad.com/en-us/prime-pcr-assays/select-plate-template).

No.	Gene symbol	Gene name	Assay ID	Design
1	CAMK2B	calcium/calmodulin-dependent protein kinase II beta	qHsaCID0037770	Intron-spanning
2	CNTNAP2	contactin associated protein-like 2	qHsaCID0018467	Intron-spanning
3	CRABP2	cellular retinoic acid binding protein 2	qHsaCID0008473	Intron-spanning
4	DLG4	discs, large homolog 4	qHsaCID0010467	Intron-spanning
5	FOXP2	forkhead box P2	qHsaCED0037795	Exonic
6	GABBR1	gamma-aminobutyric acid (GABA) B receptor, 1	qHsaCID0038341	Intron-spanning
7	GADPH	glyceraldehyde-3-phosphate dehydrogenase	qHsaCED0038674	Exonic
8	GRIA2	glutamate receptor, ionotropic, AMPA 2	qHsaCID0009010	Intron-spanning
9	H3F3B	H3 histone, family 3B (H3.3B)	qHsaCED0023847	Exonic
10	MAP2	microtubule-associated protein 2	qHsaCID0017831	Intron-spanning
11	NES	nestin	qHsaCED0001303	Exonic
12	NGFR	nerve growth factor receptor	qHsaCID0014020	Intron-spanning
13	NLGN3	neuroligin 3	qHsaCID0014894	Intron-spanning
14	NRXN1	neurexin 1	qHsaCED0037578	Exonic
15	RELN	reelin	qHsaCID0021778	Intron-spanning
16	RPL13	ribosomal protein L13	qHsaCED0001768	Exonic
17	S100B	S100 calcium binding protein B	qHsaCED0003330	Exonic
18	SEMA5A	Semaphorin 5A	qHsaCID0036421	Intron-spanning
19	SHANK1	SH3 and multiple ankyrin repeat domains 1	qHsaCID0009046	Intron-spanning
20	SLC6A4	solute carrier family 6 (neurotransmitter transporter, serotonin), member 4	qHsaCID0016255	Intron-spanning
21	SYP	synaptophysin	qHsaCED0002771	Exonic
22	TPH2	tryptophan hydroxylase 2	qHsaCID0008137	Intron-spanning
23	UNC5B	unc-5 homolog B (C. elegans)	qHsaCID0021074	Intron-spanning

Supplementary Table S2. Summary of the function of the genes selected for RT-qPCR and their known association with developmental disorders.

Genes	Functions	potential association with neurodevelopmental disorders	References
CAMK2B	Synaptic plasticity	Intellectual disability	[1,2]
DLG4	Synaptic regulation	Synaptopathy	[3]
MAP2	Neurite outgrowth	ASD	[4,5]
CRABP2	RA signaling pathway	Motor delay, CNS anomalies	[6]
FOXP2		Speech disorders, ASD	[7]
NES	Neuronal development/differentiation		[8]
GABRR1	GABA receptor	ASD	[9,10]
CNTNAP2		Language disability, ASD	[11]
NGFR	neurogenesis		[12]
UNC5B	Blood-brain barrier integrity, axon development		[13,14]
GRIA2	Synaptic transmission	Intellectual disability	[15,16]
RELN	Neuronal migration and maturation	Intellectual disability, ASD	[17,18]
NLGN3	Neurite outgrowth	ASD	[19-21]
NRXN1	Synaptic signaling	ASD	[22,23]
SHANK1	Synaptic formation and development	ASD	[24,25]
S100B	Calcium binding protein, astrocyte marker	ASD	[26,27]
SEMA5A	Axonal guidance	ASD	[28]
TPH2	Neurotransmission	ASD	[29]
SLC6A4	Neurogenesis, synaptic plasticity	ADHD	[30-32]
SYP	Synaptic plasticity	Intellectual disability	[33-35]

References

1. Borghi, R.; Trivisano, M.; Specchio, N.; Tartaglia, M.; Compagnucci, C. Understanding the pathogenetic mechanisms underlying altered neuronal function associated with CAMK2B mutations. *Neurosci. Biobehav. Rev.* **2023**, *152*, 105299. https://doi.org/10.1016/j.neubiorev.2023.105299.
2. Nicole, O.; Pacary, E. CaMKIIβ in Neuronal Development and Plasticity: An Emerging Candidate in Brain Diseases. *Int. J. Mol. Sci.* **2020**, *21*, 7272. https://doi.org/10.3390/ijms21197272.
3. Rodríguez-Palmero, A.; Boerrigter, M.M.; Gómez-Andrés, D.; Aldinger, K.A.; Marcos-Alcalde, Í.; Popp, B.; Everman, D.B.; Lovgren, A.K.; Arpin, S.; Bahrambeigi, V.; et al. DLG4-related synaptopathy: A new rare brain disorder. *Genet. Med.* **2021**, *23*, 888–899. https://doi.org/10.1038/s41436-020-01075-9.
4. Jang, D.H.; Chae, H.; Kim, M. Autistic and Rett-like features associated with 2q33.3-q34 interstitial deletion. *Am. J. Med. Genet. Part A* **2015**, *167*, 2213–2218. https://doi.org/10.1002/ajmg.a.37119.
5. Westphal, D.S.; Andres, S.; Makowski, C.; Meitinger, T.; Hoefele, J. MAP2—A Candidate Gene for Epilepsy, Developmental Delay and Behavioral Abnormalities in a Patient With Microdeletion 2q34. *Front. Genet.* **2018**, *9*, 99. https://doi.org/10.3389/fgene.2018.00099.
6. Kooblall, K.G.; Stevenson, M.; Heilig, R.; Stewart, M.; Wright, B.; Lockstone, H.; Buck, D.; Fischer, R.; Wells, S.; Lines, K.E.; et al. Identification of cellular retinoic acid binding protein 2 (CRABP2) as downstream target of nuclear factor I/X (NFIX): Implications for skeletal dysplasia syndromes. *JBMR Plus* **2024**, *8*, ziae060. https://doi.org/10.1093/jbmrpl/ziae060.
7. Reuter, M.S.; Riess, A.; Moog, U.; Briggs, T.A.; Chandler, K.E.; Rauch, A.; Stampfer, M.; Steindl, K.; Gläser, D.; Joset, P.; et al. FOXP2 variants in 14 individuals with developmental speech and language disorders broaden the mutational and clinical spectrum. *J. Med. Genet.* **2017**, *54*, 64–72. https://doi.org/10.1136/jmedgenet-2016-104094.
8. Suzuki, S.; Namiki, J.; Shibata, S.; Mastuzaki, Y.; Okano, H. The neural stem/progenitor cell marker nestin is expressed in proliferative endothelial cells, but not in mature vasculature. *J. Histochem. Cytochem. Off. J. Histochem. Soc.* **2010**, *58*, 721–730. https://doi.org/10.1369/jhc.2010.955609.
9. Zhu, F.; Feng, M.; Sinha, R.; Murphy, M.P.; Luo, F.; Kao, K.S.; Szade, K.; Seita, J.; Weissman, I.L. The GABA receptor GABRR1 is expressed on and functional in hematopoietic stem cells and megakaryocyte progenitors. *Proc. Natl. Acad. Sci. USA* **2019**, *116*, 18416–18422. https://doi.org/10.1073/pnas.1906251116.
10. Blatt, G.J.; Fatemi, S.H. Alterations in GABAergic Biomarkers in the Autism Brain: Research Findings and Clinical Implications. *Anat. Rec.* **2011**, *294*, 1646–1652. https://doi.org/10.1002/ar.21252.

11. Agarwala, S.; Ramachandra, N.B. Role of CNTNAP2 in autism manifestation outlines the regulation of signaling between neurons at the synapse. *Egypt. J. Med. Hum. Genet.* **2021**, *22*, 22. https://doi.org/10.1186/s43042-021-00138-z.
12. Siddiqui, T.; Cosacak, M.I.; Popova, S.; Bhattarai, P.; Yilmaz, E.; Lee, A.J.; Min, Y.; Wang, X.; Allen, M.; İş, Ö.; et al. Nerve growth factor receptor (Ngfr) induces neurogenic plasticity by suppressing reactive astroglial Lcn2/Slc22a17 signaling in Alzheimer's disease. *npj Regen. Med.* **2023**, *8*, 33. https://doi.org/10.1038/s41536-023-00311-5.
13. de Leeuw, V.C.; van Oostrom, C.T.M.; Wackers, P.F.K.; Pennings, J.L.A.; Hodemaekers, H.M.; Piersma, A.H.; Hessel, E.V.S. Neuronal differentiation pathways and compound-induced developmental neurotoxicity in the human neural progenitor cell test (hNPT) revealed by RNA-seq. *Chemosphere* **2022**, *304*, 135298. https://doi.org/10.1016/j.chemosphere.2022.135298.
14. Boyé, K.; Geraldo, L.H.; Furtado, J.; Pibouin-Fragner, L.; Poulet, M.; Kim, D.; Nelson, B.; Xu, Y.; Jacob, L.; Maissa, N.; et al. Endothelial Unc5B controls blood-brain barrier integrity. *Nat. Commun.* **2022**, *13*, 1169. https://doi.org/10.1038/s41467-022-28785-9.
15. Salpietro, V.; Dixon, C.L.; Guo, H.; Bello, O.D.; Vandrovcova, J.; Efthymiou, S.; Maroofian, R.; Heimer, G.; Burglen, L.; Valence, S.; et al. AMPA receptor GluA2 subunit defects are a cause of neurodevelopmental disorders. *Nat Commun* **2019**, *10*, 3094. https://doi.org/10.1038/s41467-019-10910-w.
16. Coombs, I.D.; Ziobro, J.; Krotov, V.; Surtees, T.-L.; Cull-Candy, S.G.; Farrant, M. A gain-of-function GRIA2 variant associated with neurodevelopmental delay and seizures: Functional characterization and targeted treatment. *Epilepsia* **2022**, *63*, e156–e163. https://doi.org/10.1111/epi.17419.
17. Huang, C.-C.; D'Arcangelo, G. The Reelin Gene and Its Functions in Brain Development. In *Reelin Glycoprotein: Structure, Biology and Roles in Health and Disease*, Fatemi, S.H., Ed.; Springer New York: New York, NY, USA, 2008; pp. 1–13. https://doi.org/10.1007/978-0-387-76761-1_1.
18. Joly-Amado, A.; Kulkarni, N.; Nash, K.R. Reelin Signaling in Neurodevelopmental Disorders and Neurodegenerative Diseases. *Brain Sci.* **2023**, *13*, 1479. https://doi.org/10.3390/brainsci13101479.
19. Qin, L.; Liu, Z.; Guo, S.; Han, Y.; Wang, X.; Ren, W.; Chen, J.; Zhen, H.; Nie, C.; Xing, K.-K.; et al. Astrocytic Neuroligin-3 influences gene expression and social behavior, but is dispensable for synapse number. *Mol. Psychiatry* **2024**, 1–13. https://doi.org/10.1038/s41380-024-02659-6.
20. Bay, H.; Haghighatfard, A.; Karimipour, M.; Seyedena, S.Y.; Hashemi, M. Expression alteration of Neuroligin family gene in attention deficit and hyperactivity disorder and autism spectrum disorder. *Res. Dev. Disabil.* **2023**, *139*, 104558. https://doi.org/10.1016/j.ridd.2023.104558.
21. Xu, J.; Du, Y.-l.; Xu, J.-w.; Hu, X.-g.; Gu, L.-f.; Li, X.-m.; Hu, P.-h.; Liao, T.-l.; Xia, Q.-q.; Sun, Q.; et al. Neuroligin 3 Regulates Dendritic Outgrowth by Modulating Akt/mTOR Signaling. *Front. Cell. Neurosci.* 2019, 13, 518. https://doi.org/10.3389/fncel.2019.00518.
22. Onay, H.; Kacamak, D.; Kavasoglu, A.N.; Akgun, B.; Yalcinli, M.; Kose, S.; Ozbaran, B. Mutation analysis of the NRXN1 gene in autism spectrum disorders. *Balk. J. Med. Genet. BJMG* **2016**, *19*, 17–22. https://doi.org/10.1515/bjmg-2016-0031.
23. Südhof, T.C. Neuroligins and neurexins link synaptic function to cognitive disease. *Nature* **2008**, *455*, 903–911. https://doi.org/10.1038/nature07456.
24. Qin, Y.; Du, Y.; Chen, L.; Liu, Y.; Xu, W.; Liu, Y.; Li, Y.; Leng, J.; Wang, Y.; Zhang, X.-Y.; et al. A recurrent SHANK1 mutation implicated in autism spectrum disorder causes autistic-like core behaviors in mice via downregulation of mGluR1-IP3R1-calcium signaling. *Mol. Psychiatry* **2022**, *27*, 2985–2998. https://doi.org/10.1038/s41380-022-01539-1.
25. Sala, C.; Vicidomini, C.; Bigi, I.; Mossa, A.; Verpelli, C. Shank synaptic scaffold proteins: Keys to understanding the pathogenesis of autism and other synaptic disorders. *J. Neurochem.* **2015**, *135*, 849–858. https://doi.org/10.1111/jnc.13232.
26. Tomova, A.; Keményová, P.; Filčíková, D.; Szapuová, Ž.; Kováč, A.; Babinská, K.; Ostatníková, D. Plasma levels of glial cell marker S100B in children with autism. *Physiol. Res.* **2019**, *68*, S315–s323. https://doi.org/10.33549/physiolres.934350.
27. Abboud, T.; Rohde, V.; Mielke, D. Mini review: Current status and perspective of S100B protein as a biomarker in daily clinical practice for diagnosis and prognosticating of clinical outcome in patients with neurological diseases with focus on acute brain injury. *BMC Neurosci.* **2023**, *24*, 38. https://doi.org/10.1186/s12868-023-00807-2.
28. Melin, M.; Carlsson, B.; Anckarsater, H.; Rastam, M.; Betancur, C.; Isaksson, A.; Gillberg, C.; Dahl, N. Constitutional downregulation of SEMA5A expression in autism. *Neuropsychobiology* **2006**, *54*, 64–69. https://doi.org/10.1159/000096040.
29. Yang, S.Y.; Yoo, H.J.; Cho, I.H.; Park, M.; Kim, S.A. Association with tryptophan hydroxylase 2 gene polymorphisms and autism spectrum disorders in Korean families. *Neurosci. Res.* **2012**, *73*, 333–336. https://doi.org/10.1016/j.neures.2012.05.012.
30. Veenstra-VanderWeele, J.; Muller, C.L.; Iwamoto, H.; Sauer, J.E.; Owens, W.A.; Shah, C.R.; Cohen, J.; Mannangatti, P.; Jessen, T.; Thompson, B.J.; et al. Autism gene variant causes hyperserotonemia, serotonin receptor hypersensitivity, social impairment and repetitive behavior. *Proc. Natl. Acad. Sci. USA* **2012**, *109*, 5469–5474, doi:doi:10.1073/pnas.1112345109.
31. Dark, C.; Homman-Ludiye, J.; Bryson-Richardson, R.J. The role of ADHD associated genes in neurodevelopment. *Dev. Biol.* **2018**, *438*, 69–83. https://doi.org/10.1016/j.ydbio.2018.03.023.
32. Gizer, I.R.; Ficks, C.; Waldman, I.D. Candidate gene studies of ADHD: A meta-analytic review. *Hum. Genet.* **2009**, *126*, 51–90. https://doi.org/10.1007/s00439-009-0694-x.
33. Harper, C.B.; Mancini, G.M.S.; van Slegtenhorst, M.; Cousin, M.A. Altered synaptobrevin-II trafficking in neurons expressing a synaptophysin mutation associated with a severe neurodevelopmental disorder. *Neurobiol. Dis.* **2017**, *108*, 298–306. https://doi.org/10.1016/j.nbd.2017.08.021.
34. Michetti, C.; Falace, A.; Benfenati, F.; Fassio, A. Synaptic genes and neurodevelopmental disorders: From molecular mechanisms to developmental strategies of behavioral testing. *Neurobiol. Dis.* **2022**, *173*, 105856. https://doi.org/10.1016/j.nbd.2022.105856.
35. Shen, Y.-C.; Tsai, H.-M.; Ruan, J.-W.; Liao, Y.-C.; Chen, S.-F.; Chen, C.-H. Genetic and functional analyses of the gene encoding synaptophysin in schizophrenia. *Schizophr. Res.* **2012**, *137*, 14–19. https://doi.org/10.1016/j.schres.2012.01.028.

Supplementary Figure S1. Caspase activity assay showed increased caspase activation in response to FLX treatment at 10 μM and 20 μM compared to the control demonstrating a concentration-dependent increase in caspase activity with FLX treatment after 96 h (Data represent the average of each group, derived from a single experiment with three technical replicates)

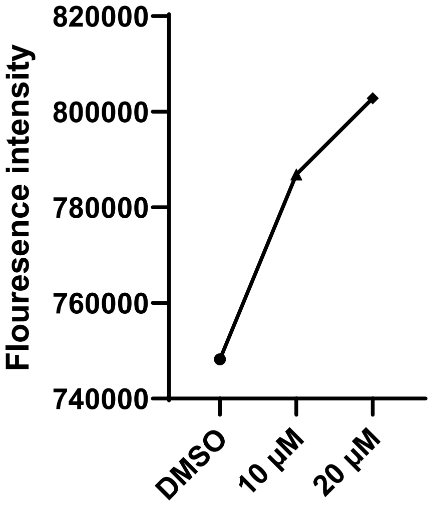

Supplement: Supplementary file 1 [file pharmaceuticals-17-01392-s001.zip › pharmaceuticals-3221211-supplementary.pdf]
